# Mechanism Failures in Externally Controlled Motorized Intramedullary Lengthening Nails: A Systematic Review

**DOI:** 10.2106/JBJS.OA.25.00327

**Published:** 2026-03-24

**Authors:** Egor Kostin, Petros Kitsis, Charalampos Charalampidis, Ioannis Orfanos

**Affiliations:** 1 Medical School, University of Cyprus, Aglantzia, Nicosia, Cyprus; 2 Department of Orthopaedic Surgery, Nicosia General Hospital, Strovolos, Nicosia, Cyprus

## Abstract

**Background:**

Externally controlled intramedullary lengthening nails (IMNs) have reduced the soft-tissue morbidity associated with external fixation. However, these complex devices introduce specific risks related to their motorized and telescopic mechanisms. Existing literature often conflates structural fatigue fractures with internal mechanism dysfunction. This systematic review aims to analyze the incidence, etiology, and management of intrinsic mechanism failures in lengthening IMNs.

**Methods:**

A comprehensive search of PubMed, Embase, and Scopus was conducted from January 2000 to July 2025 in accordance with Preferred Reporting Items for Systematic Reviews and Meta-Analyses (PRISMA) 2020 guidelines. The review included human studies reporting mechanism-related failures (e.g., jamming, backtracking, and motor failure) in externally controlled IMNs. Studies describing only structural fatigue fractures or nonexternally controlled devices were excluded (e.g., Intramedullary Skeletal Kinetic Distractor [ISKD]). Data were stratified by implant generation and material.

**Results:**

Twenty-nine studies encompassing 2,495 nails were included. The overall reported mechanism-related failure rate was 4.3% (n = 107/2,495). Distinct failure phenotypes emerged based on the implant material. Titanium-alloy nails (PRECICE P1/P2) demonstrated a 4.2% failure rate (n = 74/1,761), predominantly characterized by mechanical jamming or gear slippage due to load-induced yield. Stainless steel nails (STRYDE) exhibited a significantly higher failure rate of 12.9% (n = 12/93), primarily driven by tribocorrosion and biological reactions at the telescopic junction. The FITBONE system had a reported failure rate of 3.3% (n = 21/641). Management required surgical intervention in 97.2% of failure cases, with exchange nailing being the primary salvage strategy (94.4%). Despite the need for reoperation, the target limb length was reportedly achieved in the majority of studies where quantitative outcomes were specified.

**Conclusion:**

Mechanism failure in externally controlled lengthening IMNs is a clinically significant complication with a reported incidence of approximately 1 in 24 cases. A material trade-off is evident: Titanium implants are susceptible to mechanical gear yield, whereas stainless steel implants are prone to tribocorrosion-induced failure. While these failures necessitate revision surgery, they typically do not preclude successful limb reconstruction if managed with timely nail exchange.

**Level of Evidence:**

Level III. See Instructions for Authors for a complete description of levels of evidence.

## Introduction

Distraction osteogenesis has revolutionized the treatment of lower limb length discrepancies of bone defects. While external fixation (monoliteral, circular, etc.) was historically the standard, it is associated with high rates of pin-site infection, soft-tissue tethering, and psychological burden^1-3^. The evolution of internal limb lengthening techniques has significantly advanced patient care, particularly with utilization of motorized intramedullary nails (IMNs), which offer a more comfortable and cosmetically favorable alternative. These devices eliminate the need for external hardware, reducing many of the soft-tissue complications associated with external fixation^1^.

Despite these advantages, lengthening IMNs have their complications. Device-specific complications, such as motor dysfunction or backtracking, may necessitate revision surgeries and negatively affect patient outcomes. A comprehensive understanding of such device-related failures is essential for orthopaedic surgeons to select the most reliable implant, minimize surgical interventions, and preserve patient’s quality of life.

Although various case series and systematic reviews have addressed anatomical and patient-related complications of limb lengthening procedures, there is a notable gap in the literature regarding device-related failures specific to these devices^4-8^. Currently, no single resource offers surgeons a consolidated understanding of failure risks that are beyond their control—particularly those intrinsic to the device’s motorized and telescopic mechanisms. To address this gap, we conducted a systematic review of the literature focusing exclusively on mechanical and device-related complications associated with intramedullary lengthening nails.

## Materials and Methods

### Search Strategy

The research question, as well as inclusion and exclusion criteria for individual studies, was established before beginning the searches. Two reviewers (E.K. and I.O.) independently searched 3 online databases, PubMed, Embase, and Scopus, for the literature related to device failure in intramedullary lengthening nails. Keywords used were a combination of controlled vocabulary and free-text terms: (“magnetic nail” OR “telescopic nail” OR “PRECICE” OR “STRYDE” OR “MAGNUS”) AND (“intramedullary” OR “lengthening nail”) AND (“mechanical failure” OR “complication” OR “shortening” OR “backtracking” OR “corrosion” OR “motor failure” OR “implant failure”). The search was conducted from January 2000 to July 27, 2025, without any language restrictions. The systematic review was performed in accordance with the PRISMA 2020 guidelines.

### Inclusion and Exclusion Criteria

The following inclusion criteria were used: (1) human studies involving externally controlled intramedullary lengthening nails (e.g., PRECICE, STRYDE, and MAGNUS); (2) studies reporting mechanism-related device failures (e.g., jamming, motor failure, and backtracking); (3) all age groups and sexes, with no limitation; and (4) study designs: randomized controlled trials, prospective or retrospective cohort studies, and case series involving ≥3 patients.

The exclusion criteria were (1) studies of nonexternally controlled mechanical intramedullary lengthening systems (e.g., ISKD); (2) studies focused exclusively on structural fatigue fracture (shell breakage) unrelated to the nail mechanism; (3) case reports, cadaveric or animal studies, and biomechanical-only investigations; and (4) studies that did not provide mechanism-related outcomes of the surgical treatment.

We excluded cases of pure structural fatigue fracture (nail shaft breakage or bending or screw breakage) that occurred without prior mechanism dysfunction. This exclusion was applied to distinguish intrinsic mechanism reliability (a device engineering issue) from structural survivorship (often influenced by patient weight-bearing compliance and implant diameter). Our analysis focused strictly on the reliability of the actuation engine.

## Study Selection

**Fig. 1:**
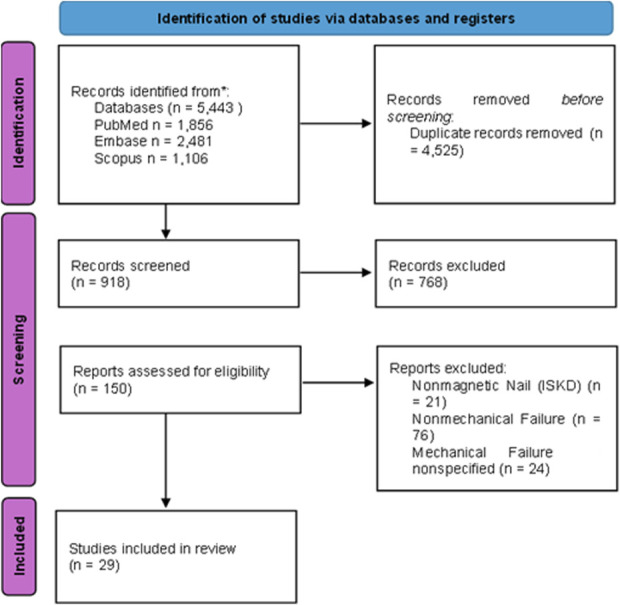
PRISMA flow diagram.

Two independent reviewers screened the titles and abstracts of the identified articles as well as potentially eligible articles in the reference lists of those articles. They then performed a full-text review of the articles that appeared to be relevant. To mitigate exclusion bias, both reviewers had independent authority to include studies for full-text review. The initial comprehensive search yielded 5,443 articles; after removal of duplicates, 918 studies remained for title and abstract screening. After initial screening, 150 full-text articles were assessed for eligibility. A total of 29 studies satisfied the inclusion and exclusion criteria. Discrepancies during screening were resolved by consensus. The remaining 29 studies were included in this systematic review (Fig. 1).

### Data Abstraction

The abstracted data included study and publication information (first author, year of publication, study design, and sample size [patients/nails]), implant characteristics (bone involved, type of lengthening nail, and material), and complication data. Specifically, we extracted the type and number of mechanism-related complications, the rate and timing of failures, and surgical details (type of reoperation). Relevant contextual notes (e.g., specific failure mode, manufacturer notes, recall status, and distraction amount) were also abstracted (if it was declared).

**Fig. 2:**
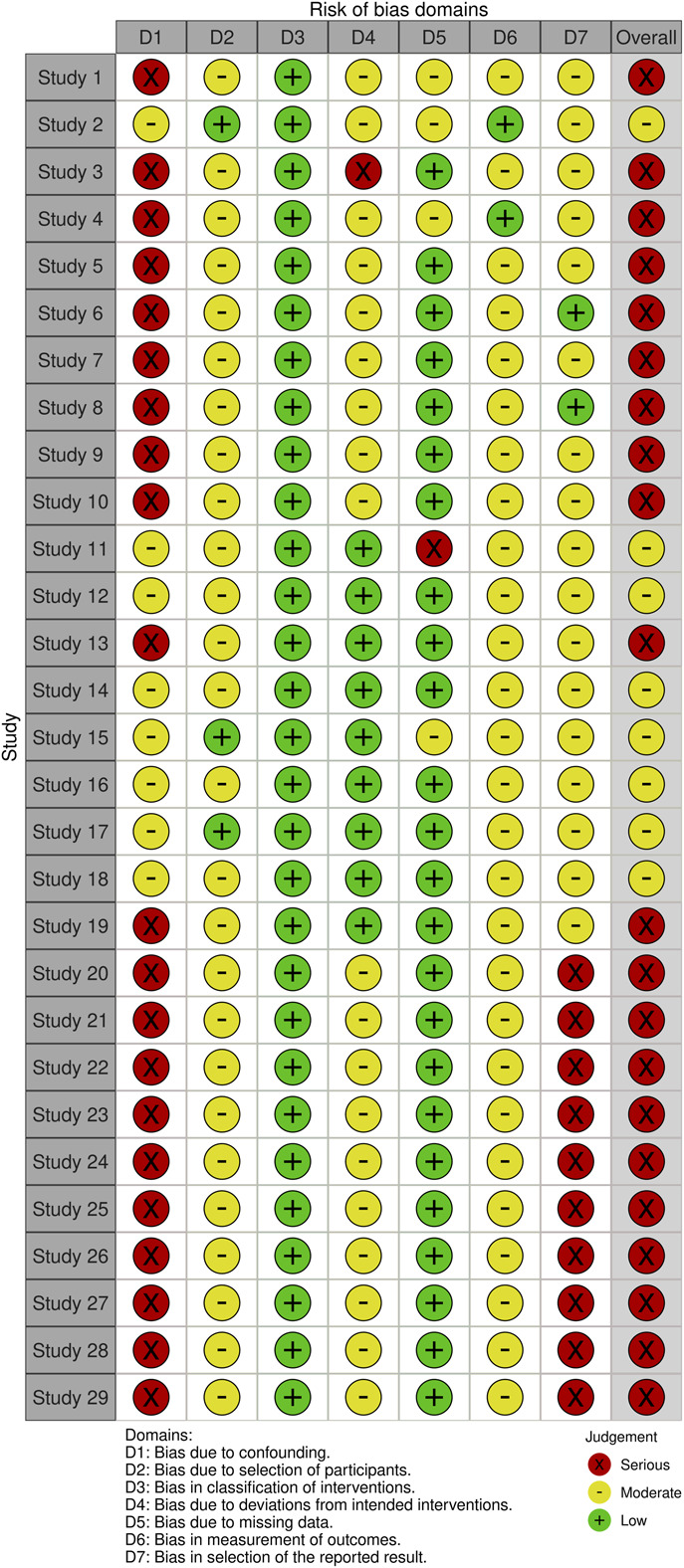
ROBINS-I traffic light plot.

**Fig. 3:**
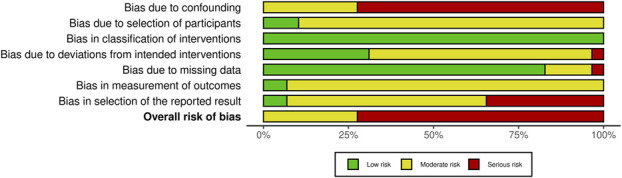
ROBINS-I summary plot.

The methodological quality of the included articles was assessed using the ROBINS-I tool (Figs. 2 and 3). Each study was assessed across 7 domains, including confounding and participant selection, with the overall risk of bias determined by the most severe domain.

## Results

A total of 29 studies were included in the review (Table I)^5,9-36^. Based on the data reported, the analysis encompassed a total of 2,495 intramedullary lengthening nails. The majority of implants were used for femoral lengthening, followed by tibial lengthening. The included devices represented the evolution of intramedullary lengthening technology: The titanium-alloy PRECICE system (P1 and P2 models) was the most frequently reported implant, followed by the stainless steel STRYDE nail and the FITBONE system. The study designs were predominantly retrospective cohort studies and case series, with publication dates ranging from 2006 to 2025.

**Table I.** Summary of Included Studies and Extracted Device-Related Failure Data^5,9-35^

Study	Link	Year	Study Type	N (Patients/Nails)	Bone (Femur/Tibia)	Nail Type	No. of Failures	Rate (%)	Type of Failure	Reoperation Needed	Notes
Clinical and radiological outcomes of magnetically controlled intramedullary lengthening nails in adolescents and young adults: a retrospective cohort study	https://pmc.ncbi.nlm.nih.gov/articles/PMC12168351	2025	Retrospective cohort study	24	Femur	PRECICE 2	1	4.17%	Distraction failure (jamming)	Nail exchange	
Bone Healing Index and complications of a magnetic internal lengthening nail: a retrospective series of 286 bone lengthening events	https://www.researchgate.net/publication/392332626_Bone_Healing_Index_and_Complications_of_a_Magnetic_Internal_Lengthening_Nail_A_Retrospective_Series_of_286_Bone_Lengthening_Events#fullTextFileContent	2025	Retrospective review	286	Femur/tibia	FITBONE	13	4.50%	Distraction failure (jamming)	Nail exchange	
Magnetically driven antegrade intramedullary lengthening nails for tibial lengthening	https://www.researchgate.net/publication/378639921_Magnetically_driven_antegrade_intramedullary_lengthening_nails_for_tibial_lengthening#fullTextFileContent	2024	Retrospective cohort study	58/67	Tibia	PRECICE	2	3.00%	Distraction failure (jamming)	Nail exchange and reosteotomy	
Simultaneous correction of leg length discrepancy and angular deformity of the distal femur with retrograde PRECICE nails: a retrospective analysis of 45 patients	https://actaorthop.org/actao/article/view/40947/46551	2024	Retrospective cohort study	45	Femur	PRECICE 1 and PRECICE 2	1	2.22%	Distraction failure (jamming)	Nail exchange and reosteotomy	Patients with preexisting deformities (flexion, varus, and valgus)
A clinical and radiological matched-pair analysis of patients treated with the PRECICE and STRYDE magnetically driven motorized intramedullary lengthening nails	https://pubmed.ncbi.nlm.nih.gov/36587248	2023	Retrospective cohort study	124/140 (49 STRYDE, 91 PRECICE)	Femur/tibia	STRYDE and PRECICE	PRECICE = 8, STRYDE = 9	PRECICE = 5.71 (9%), STRYDE = 6.43 (19%)	Distraction failure (jamming)	Nail exchange	
Complications and risk factors of intramedullary bone lengthening nails: a retrospective multicenter cohort study of 314 FITBONE and PRECICE nails	https://pmc.ncbi.nlm.nih.gov/articles/PMC9940487	2023	Retrospective review	257/314 (FITBONE = 234, PRECICE = 80)	Femur/tibia	FITBONE and PRECICE	PRECICE = 22	7.00%	Distraction failure (jamming)	Nail exchange and reosteotomy	
Complications after intramedullary fixation treatment of patients with osteogenesis imperfecta: telescopic versus nontelescopic implants	https://pmc.ncbi.nlm.nih.gov/articles/PMC10507367	2023	Retrospective cohort study	14	Femur	STRYDE	3	21.00%	Distraction failure (jamming)	Nail exchange	
Mechanical failures in magnetic intramedullary lengthening nails	https://www.researchgate.net/publication/367244761_Mechanical_Failures_in_Magnetic_Intramedullary_Lengthening_Nails	2022	Retrospective cohort study	377/420	Femur/tibia	PRECICE 1 and PRECICE 2	P1 = 5, P2 = 5	P1 = 1.19 (5.15%), P2 = 1.19 (1.54%)	P1 (backtracking = 1, jamming = 4), P2 (backtracking = 1, jamming = 4)	Nail exchange to P2	
Two-stage bone lengthening with reuse of a single intramedullary telescopic nail in patients with achondroplasia	https://journals.lww.com/pedorthopaedics/abstract/2022/07000/two_stage_bone_lengthening_with_reuse_of_a_single.18.aspx	2022	Retrospective cohort study	9/26	Femur/tibia	PRECICE	2	7.70%	Distraction failure (jamming)	Nail exchange in 1 patient and conversion to external fixation in other	
Bone lengthening with magnetic nails. Experience in patients younger than 18	https://www.sciencedirect.com/science/article/pii/S1888441521000989?via%3Dihub	2022	Retrospective cohort study	28/31	Femur/tibia	PRECICE 2	1	3.20%	Distraction failure (jamming)	Generator exchange	
Motorized intramedullary nail lengthening in the older population	https://www.mdpi.com/2077-0383/11/17/5242	2022	Retrospective case-control series	259/354	Femur/tibia	PRECICE	5	1.40%	Backtracking = 4, jamming = 1	Nail exchange	
STRYDE versus PRECICE magnetic internal lengthening nail for femur lengthening	https://pmc.ncbi.nlm.nih.gov/articles/PMC9596511	2021	Retrospective cohort study	66 (30 S & 36 P)	Femur	STRYDE and PRECICE	PRECICE = 3, STRYDE = 0	4.55 (8.33%)	Distraction failure (jamming)	Nail exchange	
Efficacy of PRECICE nail in treatment of adult patients with posttraumatic femoral leg length discrepancy	https://www.reachyourheight.com/wp-content/uploads/2022/01/2021-Brinker-Efficacy-Precice-Nail-Femoral-Leg-Length-JOrthopTraum.pdf	2021	Prospective study	29	Femur/tibia	PRECICE	1	3.49%	Distraction failure (jamming)	Exchange of controller	
PRECICE intramedullary nail in the treatment of adult leg length discrepancy	https://www.injuryjournal.com/article/S0020-1383(20)30226-6/abstract	2020	Prospective study	21	Femur/tibia	PRECICE	1	4.76%	Distraction failure (jamming)	Nail exchange	
How should we lengthen posttraumatic limb defects? A systematic review and comparison of motorized lengthening systems, combined internal and external fixation and external fixation alone	https://link.springer.com/article/10.1007/s00590-020-02831-y	2020	Systematic review	87	Femur/tibia	PRECICE	2	2.30%	Distraction failure (jamming)	Nail exchange	
Lengthening of the humerus using a motorized lengthening nail: a retrospective comparative series	https://sci-hub.se/10.1097/BPO.0000000000001453	2020	Retrospective cohort study	6	Humerus	PRECICE	1	16.70%	Distraction failure (jamming)	Nail recharge	
Femoral lengthening using the PRECICE intramedullary limb-lengthening system	https://sci-hub.se/10.1302/0301-620X.101B9.BJJ-2018-1271.R1	2019	Retrospective review of prospectively collected data	92/107	Femur	PRECICE	2	1.86%	Backtracking = 1, jamming = 1	Nail exchange	
Limb lengthening and deformity correction with externally controlled motorized intramedullary nails: evaluation of 50 consecutive lengthenings	https://pmc.ncbi.nlm.nih.gov/articles/PMC6366464	2018	Retrospective review	47/50 (34 = PRECICE, 16 = FITBONE)	Femur	PRECICE and FITBONE	PRECICE = 1	2 (4.8%)	Distraction failure (jamming)	Nail exchange	
A comparison of the device-related complications of intramedullary lengthening nails using a new classification system	https://pmc.ncbi.nlm.nih.gov/articles/PMC5654310	2017	Prospective study	60/115	Femur/tibia	PRECICE 1 and PRECICE 2	P2 = 2	P2 = 4.4 (1.74%)	P2 (backtracking = 1, jamming = 1)	Nail exchange	
Evaluation of the first experience of intramedullary nail lengthening using PRECICE® in a South African limb lengthening and reconstruction unit	https://www.scielo.org.za/pdf/saoj/v15n1/09.pdf	2016	Retrospective cohort study	9/11	Femur	PRECICE	1	9.00%	Distraction failure (jamming)	Nail exchange + overream	
Bone lengthening using the Fitbone® motorized intramedullary nail: the first experience in France	https://www.sciencedirect.com/science/article/pii/S1877056816000062?via%3Dihub	2016	Prospective study	23/26	Femur	FITBONE	1	3.84%	Distraction failure (jamming)	Nail exchange	
Pitfalls in automatic limb lengthening—first results with an intramedullary lengthening device	https://www.sciencedirect.com/science/article/pii/S1877056816300883?via%3Dihub	2016	Retrospective cohort study	10	Femur	PRECICE	2	20.00%	Control failure (backtracking)	Nail exchange	
Stature lengthening using the PRECICE intramedullary lengthening nail	https://sci-hub.se/10.1097/BTO.0000000000000140	2015	Retrospective cohort study	51/116	Femur/tibia	PRECICE 1 and PRECICE 2	P1 = 3	2.6% in all nails (5% in the P1 group)	Distraction failure (jamming)	Nail exchange (+ reosteotomy in 2 case), 3rd case was not treated and therapy stopped 1 cm short	
Precision of the PRECICE® internal bone lengthening nail	https://pmc.ncbi.nlm.nih.gov/articles/PMC4397804	2014	Retrospective review	24/25	Femur	PRECICE	1	4.00%	Distraction failure (jamming)	Nail exchange	
How precise is the PRECICE compared to the ISKD in intramedullary limb lengthening?	https://pmc.ncbi.nlm.nih.gov/articles/PMC4062798	2014	Retrospective cohort study	24/26	Femur/tibia	PRECICE	2	7.70%	Distraction failure (jamming)	Nail exchange in 1 patient and conversion to other procedure in other	Osteogenesis imperfecta
Functional results of lower extremity lengthening by motorized intramedullary nails	https://www.aott.org.tr/en/functional-results-of-lower-extremity-lengthening-by-motorized-intramedullary-nails-164191	2012	Retrospective cohort study	14/15	Femur/tibia	FITBONE	2	13.30%	Distraction failure (jamming)	Conversion to lengthening with monolateral fixator over nail	
Intramedullary leg lengthening with a motorized nail	https://pmc.ncbi.nlm.nih.gov/articles/PMC3235314	2011	Retrospective cohort study	32	Femur/tibia	FITBONE	1	3.10%	Distraction failure (jamming)	Nail exchange	
Leg lengthening with a motorized nail in adolescents	https://pmc.ncbi.nlm.nih.gov/articles/PMC2505303	2008	Prospective study	8	Femur	FITBONE	2	25.00%	Distraction failure (jamming)	Nail exchange in 1 case and exchange to trauma nail in another	
The results of limb lengthening by callus distraction using an extending intramedullary nail (FITBONE) in nontraumatic disorders	https://sci-hub.se/10.1302/0301-620X.88B7.17618	2006	Retrospective cohort study	10/24	Femur/tibia	FITBONE	2	8.30%	Distraction failure (jamming)	Nail exchange + overream	Both patients are with congenital abnormalities

The mechanism-related failure rate, defined as a malfunction of the internal actuation or distraction mechanism excluding structural fatigue fractures, was 4.3% (107 of 2,495 nails). When stratified by implant generation, distinct failure patterns were observed. In the titanium (PRECICE) cohort (*n* = 1,761), the failure rate was 4.2% (*n* = 74). The primary mode of failure in this group was distraction failure (jamming) or internal gear slippage, often attributed to mechanical yield of the titanium components under load. Conversely, the stainless steel (STRYDE) cohort (*n* = 93) demonstrated a markedly higher mechanism failure rate of 12.9% (*n* = 12). In these cases, failures were characterized by tribocorrosion—a material degradation process where friction between the steel telescopic junctions releases corrosive debris, leading to biological osteolysis and mechanism seizure. The FITBONE group accounted for the remaining failures (*n* = 21), with a pooled rate of 3.3%. In this implant group, the failure phenotype was distinct from the magnetic group, reflecting its electromechanical design. Failures included subcutaneous receiver/antenna cable breakage (preventing signal transmission), motor insufficiency (where the motor lacked sufficient torque to overcome soft-tissue resistance), and mechanism jamming. In several cases, structural fatigue fracture of the nail was observed as a secondary complication following the initial jamming event.

Management of device-related failures required secondary surgical intervention in the vast majority of cases (97.2%). In all of the described failure events, the removal of the malfunctioning device was the pillar of treatment. The most common revision procedure was a single-stage exchange nailing (94.4%), where the faulty magnetic nail was replaced with either a new magnetic nail or a static trauma nail, depending on the consolidation status. Conversion to external fixation was performed in 12% of failure cases, primarily when adequate length had not yet been achieved and further internal lengthening was deemed unsafe. In the specific context of the STRYDE nail, revisions were often accompanied by the debridement (overream) of corrosion-related debris found at the telescopic interface. In the limited subset of studies reporting quantitative final outcomes, the authors reported that the final target length was eventually achieved following revision surgery. However, data regarding the exact percentage of length gained versus the original goal were frequently absent. Regarding the magnitude of distraction, 5 studies explicitly reported the total limb length achieved at the conclusion of treatment (ranging from 40 mm to 81 mm). Reporting of the specific timing of mechanism failure was also sparse, with only 4 of the 29 studies providing precise data. In these cases, failures were identified early in the distraction phase, occurring at 10, 14, 16, and 28 days postoperatively (Table I).

## Discussion

This is the first systematic review focused on mechanical device failures in intramedullary lengthening nails. While biological complications are well described, the reliability of motorized distraction systems remains less reported. By isolating actuator, telescopic, and internal mechanical failures, we highlight risks independent of surgical technique or patient factors. The principal findings of this study are threefold. First, mechanism malfunction is not rare, occurring in a reported 4.3% of nails, with 97.2% of failures necessitating operative revision (in instances where the malfunction occurred at the conclusion of treatment, some surgeons elected not to proceed with nail-exchange surgery) (Table I). Second, distinct implants demonstrate distinct failure phenotypes, suggesting that “magnetic nails” cannot be considered, a homogeneous class of technology. Third, although disruptive, these failures often allow for the achievement of planned limb length when timely salvage strategies are employed, though this conclusion is limited by the heterogeneity of outcome reporting.

### Incidence and Nature of Mechanism Failure

The observed failure rate challenges the perception that modern intramedullary nails provide predictable, maintenance-free distraction. Many published studies emphasize alignment accuracy, bone regeneration quality, and consolidation time, implicitly assuming mechanism distraction reliability. However, our findings suggest that actuator malfunction is a clinically relevant complication category that surgeons must acknowledge. Counselling patients on a 1-in-24 intrinsic implant risk parallels the paradigm in arthroplasty, where implant survivorship is an accepted part of informed consent^37-40^. Recognizing mechanism malfunction as an independent complication domain prompts re-evaluation of expectations of motorized implants as self-sufficient.

### Device Generation Differences and Failure Phenotypes

This review demonstrates implant-specific failure patterns. Titanium-based PRECICE devices exhibited predominantly mechanical failures such as jamming, internal slippage, or failure to continue distraction when mechanical yield thresholds were surpassed^41,42^. This aligns with laboratory findings indicating titanium’s susceptibility to microplastic deformation under eccentric and cyclic loading when housed in precision-machined telescopic interfaces^43,44^. By contrast, STRYDE failures most often involved corrosion or biologically mediated dysfunction at the telescopic junction. The stainless steel redesign was intended to allow early full–weight bearing, yet its steel chemistry and surface treatment appear to have facilitated a reactive intraosseous environment, later confirmed by recall investigations reporting osteolysis and metallosis^45-49^. FITBONE failures were more heterogeneous and characterized mostly by their electromechanical etiology. Unlike the fully internal magnetic drive of the PRECICE/STRYDE, the FITBONE relies on a subcutaneous receiver connected by a cable. This introduces a specific failure point: the breakage or disconnection of the antenna cable due to shear forces or fatigue. In addition, “jamming” in this cohort was frequently attributed to insufficient motor torque relative to the distraction force required, leading to distraction arrest. This contrasts with the PRECICE cohort, where jamming was typically caused by internal gear deformation or weld failure. Collectively, these findings emphasize that implant choice inherently exposes patients to different modes of failure, and ongoing postmarket surveillance remains essential for assessing reliability.

### Clinical Consequences of Failure

Management of device-related failures required secondary surgical intervention in 97.2% of cases. The most common revision procedure was a single-stage exchange nailing (94.4%). Regarding the final limb length, reporting consistency varied across the included studies. In the 5 studies that provided specific quantitative outcomes following revision, the original target length was eventually achieved in the majority of patients. However, in 1 case (Table I—line 24), the lengthening goal was modified. A surgeon elected to accept a “clinically functional” result that was 1 cm shorter than the original plan rather than pursue further high-risk interventions. In the remaining studies, final distraction amounts were not explicitly reported, limiting the ability to calculate a precise “rescue rate” for mechanism failures. This distinction is clinically important: Mechanical failure compromises the treatment trajectory but does not typically undermine the ultimate outcome.

### Implications for Practice

Several practice recommendations arise. Implant selection should be tailored to the biomechanical environment; titanium constructs may be advantageous in lower-load tibiae, whereas the reliability of stainless steel designs requires cautious interpretation until further redesign evidence is available. Routine monitoring should incorporate functional actuator assessments, as radiographic progression alone cannot confirm mechanism integrity. Early identification of dysfunction is critical, since timely exchange nailing can restore the treatment trajectory if undertaken before regenerate maturation and solidification. Collectively, these recommendations emphasize complication mitigation rather than device abandonment.

### Clinical Warning

While the metric of “goal achieved” implies success, it obscures the significant clinical and economic burden imposed by mechanism failure. Even if the final length is obtained, the “cost” of a mechanism failure involves an unplanned return to the operating room, additional anesthetic risk, prolonged latency phases, and the psychological distress of a patient. In cases of runaway nails or acute jamming, the sudden end or acceleration of distraction process can also compromise the bone regeneration quality. Therefore, surgeons must not view salvageability as a justification for device unreliability because the revision surgery is a substantial deviation from the standard of care.

## Limitations and Future Directions

This review has several limitations. Inconsistent reporting of mechanism malfunctions likely underestimates true incidence, while variable follow-up durations may obscure delayed failures. Data on newer implant generations remain insufficient, and publication bias is possible, particularly during early commercial phases. In addition, a significant limitation of this review is the likely disparity between the reported incidence of mechanism failure and the actual failure rates in clinical practice. The majority of included studies were retrospective case series, which are subject to selection and publication bias; series with favorable outcomes are more likely to be published than those with high complication rates. Furthermore, minor mechanism malfunctions—such as temporary jamming resolved by external manipulation or partial backtracking that did not necessitate reoperation—may be underreported in retrospective chart reviews. Consequently, the failure rates calculated in this review should be interpreted as a minimum baseline rather than an absolute incidence. Despite these constraints, the findings provide meaningful insights and a foundation for structured surveillance. Moving forward, research should prioritize standardized reporting frameworks with explicit documentation of actuator reliability, device-specific registries to monitor generation-dependent failures, material science investigations into corrosion and deformation-resistant systems, and exploration of biological factors influencing actuator performance. Addressing these priorities will advance limb lengthening from a technique-focused intervention to one grounded in system reliability engineering.
